# Nestin-GFP transgene labels immunoprivileged bone marrow mesenchymal stem cells in the model of ectopic foci formation

**DOI:** 10.3389/fcell.2022.993056

**Published:** 2022-09-05

**Authors:** Dmitriy Karpenko, Nikolay Kapranov, Aleksei Bigildeev

**Affiliations:** ^1^ Laboratory of Physiology of Hematopoiesis, National Medical Research Center for Hematology, Moscow, Russia; ^2^ Immunophenotyping Department, National Medical Research Center for Hematology, Moscow, Russia

**Keywords:** MSCs, Nes-Gfp, nestin-GFP, immunological surveillance, immune privilege, ectopic foci, nestin, CSCs

## Abstract

Immune privileges are demonstrated for different types of quiescent stem cells of adult mammalian organisms. Mesenchymal stem cells (MSCs) are believed to have immune privileges; however, an accurate experimental confirmation hasn’t been presented. Here, we provide direct experimental evidence that MSCs of C57Black/6J murine bone marrow (BM) are immune privileged *in vivo* and retain their functionality after prolonged exposure to the uncompromised immune system. The BM of Nes-Gfp transgenic mice was implanted as a tissue fragment under the kidney capsule in isogenic C57Black/6J immunocompetent recipients. Nestin-Gfp strain provides a fluorescent immunogenic marker for a small fraction of BM cells, including GFP^+^CD45^–^ MSCs. Despite the exposure of xenogenically marked MSCs to the fully-functional immune system, primary ectopic foci of hematopoiesis formed. Six weeks after implantation, multicolor fluorescence cytometry revealed both GFP^+^CD45^–^ and GFP^+^CD45^+^ cells within the foci. GFP^+^CD45^–^ cells proportion was 2.0 × 10^–5^ ×÷9 and it didn’t differ significantly from syngenic Nes-GFP transplantation control. According to current knowledge, the immune system of the recipients should eliminate GFP^+^ cells, including GFP^+^ MSCs. These results show that MSCs evade immunity. Primary foci were retransplanted into secondary Nes-GFP recipients. The secondary foci formed, in which CD45^–^GFP^+^ cells proportion was 6.7 × 10^–5^ ×÷2.2, and it didn’t differ from intact Nes-GFP BM. The results demonstrate that MSCs preserve self-renewal and retain their functionality after prolonged immune exposure. The success of this study relied on the implantation of BM fragments without prior dissociation of cells and the fact that the vast majority of implanted cells were immunologically equivalent to the recipients.

## Introduction

The capability to avoid immune surveillance in an organism is called immune privilege (IP). It is observed in some organs, for example, the brain, eye, meniscus, or testicles (Carson et al., 2006; Fijak and Meinhardt 2006; Stein-Streilein 2013; Smith, Costa, and Spalding 2015). The healthy functioning of immune-privileged organs could be impaired after injury or infection when integrity have been disturbed, immune barriers penetrated, and antigens presented to the immune system (Stephenson et al., 2018; Sun, Liu, and Luo 2020). IPs in tissues and organs provide suppression of inflammation and promote immune tolerance to the whole organ resulting in the protection of the whole transplant, given allogenic transplantation have been made (Streilein 2003; Hill et al., 2021). In the case of corneal transplantation, it makes the procedure the most successful among all solid organ transplantations performed in humans (Hori et al., 2019). In addition to organ level, IPs are known for individual cells both in physiological and pathological conditions (Ichiryu and Fairchild 2013). IPs can be utilized by tumor cells during oncogenesis (Joyce and Fearon 2015) and by cancer stem cells (CSCs) particularly (Malladi et al., 2016). Despite the majority of studies devoted to IPs of individual cells are focused on CSCs (Bruttel and Wischhusen 2014; Miao et al., 2019; Galassi et al., 2021), there are several studies showing IPs as a feature of normal stem cells. Early studies performed on cell cultures argued for the ability of hematopoietic stem cells (HSCs) (Aguila and Weissman 1996), embryonal stem cells (ESCs) (Drukker et al., 2002), neural stem cells (NSCs) (Mammolenti et al., 2004), and mesenchymal stem cells (MSCs) (Rasmusson et al., 2003) to evade cytotoxic action of CD8^+^ T-cells due to low MHC I type expression and simultaneously elude attack of NK-cells. More recent studies extended these observations for some types of stem cells in experiments performed *in vivo*. Studies in mice demonstrated IPs as an intrinsic property of quiescent stem cells in hair follicles (HFSCs) and muscles (MuSCs) (Agudo et al., 2018). The authors showed a reduced expression level of MHC-1, B2m, and several other genes of the antigen-presenting complex in quiescent stem cells. The authors suggested that IPs may be shared among a wide range of resting stem cells. It is known that long-term repopulating HSCs and MSCs are quiescent *in vivo* (Méndez-Ferrer et al., 2010; Bernitz et al., 2016). The immunogenicity of the total mass of stromal and hematopoietic cells of the allogenic bone marrow (BM) is beyond doubt (Ankrum, Ong, and Karp 2014; Fürst et al., 2019), but this does not exclude the possibility of the presence of minor cell populations that are immune privileged. Indeed, it was shown for HSCs, that CD150^high^ regulatory T-cells protected them from oxidative stress and kept them quiescent. At the same time, the authors showed IPs of quiescent HSCs but did not associate the dormant state and IPs as a cause and effect (Hirata et al., 2018). We show that the findings of these studies can be extrapolated to MSCs.

MSCs are thought to reside in a variety of organs and tissues (Friedenstein et al., 1987; Caplan 1991; Fraser et al., 2006; Barlow et al., 2008; Caplan 2010; de Girolamo et al., 2013; Sharpe 2016). They are responsible for the formation and maintenance of organs’ stroma. To satisfy a criterion of stem cells, they should possess specific characteristics (Horwitz et al., 2005). For example, MSCs of BM should have high proliferative potential and be able to differentiate in all the lineages of BM stromal cells, including osteogenic, adipogenic, and chondrogenic lineages to form and maintain a functional hematopoietic territory of BM. Particularly, MSCs of BM should have high proliferative potential and be able to differentiate in all the lineages of BM stromal cells, including osteogenic, adipogenic, and chondrogenic lineages to form and maintain a functional hematopoietic territory of BM (Friedenstein et al., 1970; Friedenstein et al., 1987; Caplan 1991; Weiss et al., 2010).

Using transgenic mice with GFP under nestin regulatory elements (strain Nestin-GFP) (Mignone et al., 2004), Mendez-Ferrer and colleagues showed that nestin expression characterizes MSCs, and the ability of CD45^–^Nestin^+^ cells to form a hematopoietic territory *in vivo* was demonstrated (Méndez-Ferrer et al., 2010). It was shown that CD45^–^Nestin^+^ cells contain all the BM colony-forming-unit fibroblastic (CFU-F) activity, they are spatially associated with HSCs and highly express HSC maintenance genes. CD45^–^Nestin^+^ cells regulate the resting state of HSCs being quiescent and metabolically active themselves (Méndez-Ferrer et al., 2010; Kunisaki et al., 2013). The immunogenicity of GFP provides significant technical obstacles for tracking experiments utilizing transplantation of GFP-marked cells (Ansari et al., 2016), however, the same immunogenicity coupled with reporting property of GFP can be transformed into an instrument for experiments studying immune response (Agudo et al., 2018). There are several models for studying BM stroma (Friedenstein, Chailakhjan, and Lalykina 1970; Dexter 1979; Chertkov and Gurevitch 1984; Méndez-Ferrer et al., 2010), and the models of ectopic foci of hematopoiesis formation are the most physiological ones (Tavassoli et al., 1970; Friedenstein et al., 1968; Tavassoli and Crosby 1968). These models make it possible to observe *de novo* stroma formation. The possibility of multiple sequential transfers of the hematopoietic territory has also been shown, which implicitly testified to the high proliferative potential of MSCs (Chertkov and Gurevitch 1984). So, this method is the best physiological method for determining functional MSCs today.

**FIGURE 1:**
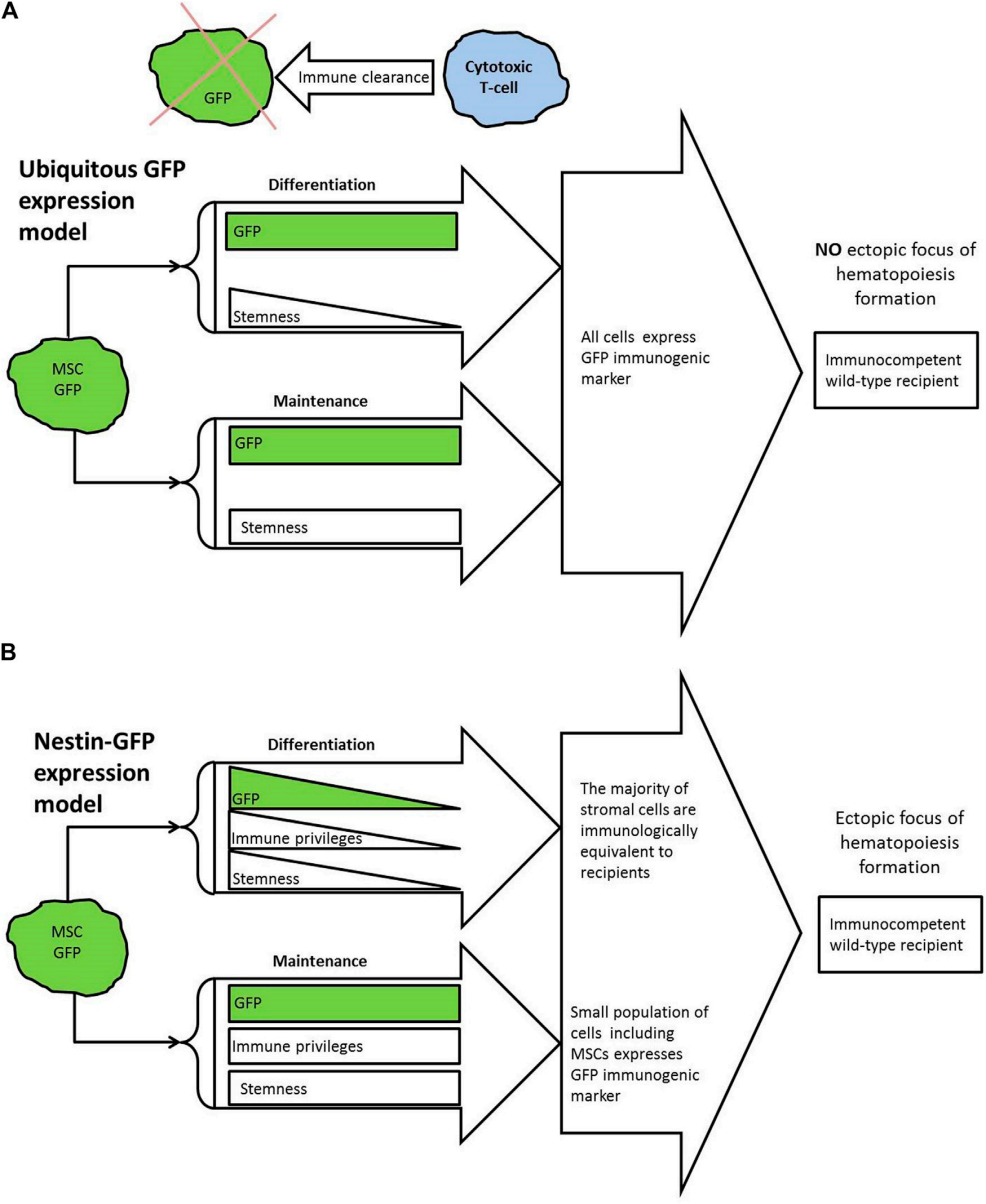
**(A)** Summary of knowledge and theoretical model. GFP is known to activate cytotoxic T-cells in wild-type recipients that results in immune clearance of GFP-marked transplants. Bone marrow stroma is known to be not immune privileged in general. So, if all implanted stromal cells express GFP, this should not lead to the formation of ectopic foci with GFP^+^ cells in immunocompetent wild-type recipient. **(B)** Overview of presented experimental model. For used Nes-GFP strain, GFP expression was demonstrated in bone marrow MSCs. GFP expression known to fade in course of differentiation. According to presented results, small part of GFP-producing cells and particularly MSCs are capable to survive in immunocompetent mice of wild-type for 6 weeks and keep their functionality. In this case GFP does not interfere with focus formation.

Having in our hands the Nestin-GFP mouse strain, in which Nes^+^ MSCs express GFP, we designed a study, in which we posed the question of what will happen to the stroma of Nestin-GFP mice when the immune system acts on it. We implanted BM of transgenic mice under the kidney capsule of mice lacking the transgene. According to current concepts, the recipient’s immune system should have destroyed GFP^+^ cells, including all Nes-GFP^+^ MSCs (Figure 1A). In this case, the formation of foci would be questionable. Nevertheless, 6 weeks after implantation of transgenic BM, normal foci of ectopic hematopoiesis were found. GFP-positive cells were detected in the foci by multicolor flow cytometry (MFC) (Figure 1B). Moreover, these cells retained their functionality, since the primary foci were able to form the secondary foci. Our results demonstrate that MSCs possess IPs *in vivo* and preserve their functionality after prolonged exposure to the whole immune system despite the presence of an immunogenic marker.

## Materials and methods

### Animals

The experiments were carried out in accordance with the United Kingdom Animals (Scientific Procedures) Act, 1986 and associated guidelines, EU Directive 2010/63/EU for animal experiments. The Commission on Biomedical Ethics of the Institute of Biomedical Problems of the Russian Academy of Sciences approved the study (Protocol No. 257). We used Nestin-GFP x C57Black/6J first generation hybrid (F1) mice of both sexes at the age 24—57 weeks, heterozygous for the eGFP transgene. Nes-GFP mice were kindly provided by prof. Grigori Enikolopov from the Research Foundation of State University of New York. We also used C57Black/6J mice of both sexes at the age 27—41 weeks. The animals were kept at the Animal Facility of the National Research Center for Hematology with a 12/12 h day/night cycle and food and water *ad libitum*.

### Foci of ectopic hematopoiesis

We used the method of ectopic foci of hematopoiesis formation (Tavassoli and Crosby 1968). The BM of one femur of an individual was used for implantation to the subcapsular region of the kidney of a recipient, as described (Chamberlin et al., 1974; Robertson, Fairchild, and Waldmann 2007; Shultz et al., 2014). Surgical manipulations were as follows. Access to the femur was opened by a skin incision on the belly next to the inner side of the hind limbs. The femoral muscles were cut off with scissors, and the diaphysis was excised, which was placed on ice until further use (up to 40 min). The recipients received anesthesia with 0.5 ml of Avertin (Sigma, United States). In recipients, the skin was cut with scissors from the back between the lower edge of the ribs and the pelvis, and the peritoneum was incised with scissors in the same place. A kidney was found in an open area with tweezers, taken out and fixed with tweezers passed under the renal vein. The kidney surface was moistened with phosphate-buffered saline (PBS) (MP Biomedicals, United States) to prevent drying. A needle with a fused end was placed into the femoral cavity at one end of the diaphysis, and the BM was squeezed out onto a dental spatula at the opposite end. The end of the spatula with the BM was placed under the kidney capsule, which had been torn with tweezers. After that, the capsule was released, which made it possible to fix the implanted BM. The kidney was released and returned into the abdominal cavity, and the peritoneum and skin were sutured with one stitch. The operation was performed in non-sterile conditions. After 42 days, each recipient was sacrificed, and the kidney was isolated with surgical instruments and placed on ice. The presence of ectopic focus of hematopoiesis was assessed. The capsule, together with the newly formed focus of hematopoiesis, was detached from the kidney. For retransplantation, an entire focus was placed under the kidney capsule of a secondary recipient as described above for transplantation. Alternatively, the surface of the kidney was washed with 1 ml of PBS. The kidney and associated connective tissues were removed. The contents of the focus (if any) were flushed out from the ossicle a few times with a gentle flow of 0.5 ml PBS using a 1 ml sampler. The samples were kept on ice until further use. The size of the formed focus of ectopic hematopoiesis was determined by the number of nuclear cells counted in the Goryaev chamber or the Abacus Junior 30 hematology analyzer (Diatron, Hungary). The osteogenic activity of stromal progenitors was assessed by the presence of the newly formed bone. The cell suspension of the focus was divided in half between samples for analysis of cell populations using MFC and for DNA isolation.

### Transplantation schemes

**FIGURE 2:**
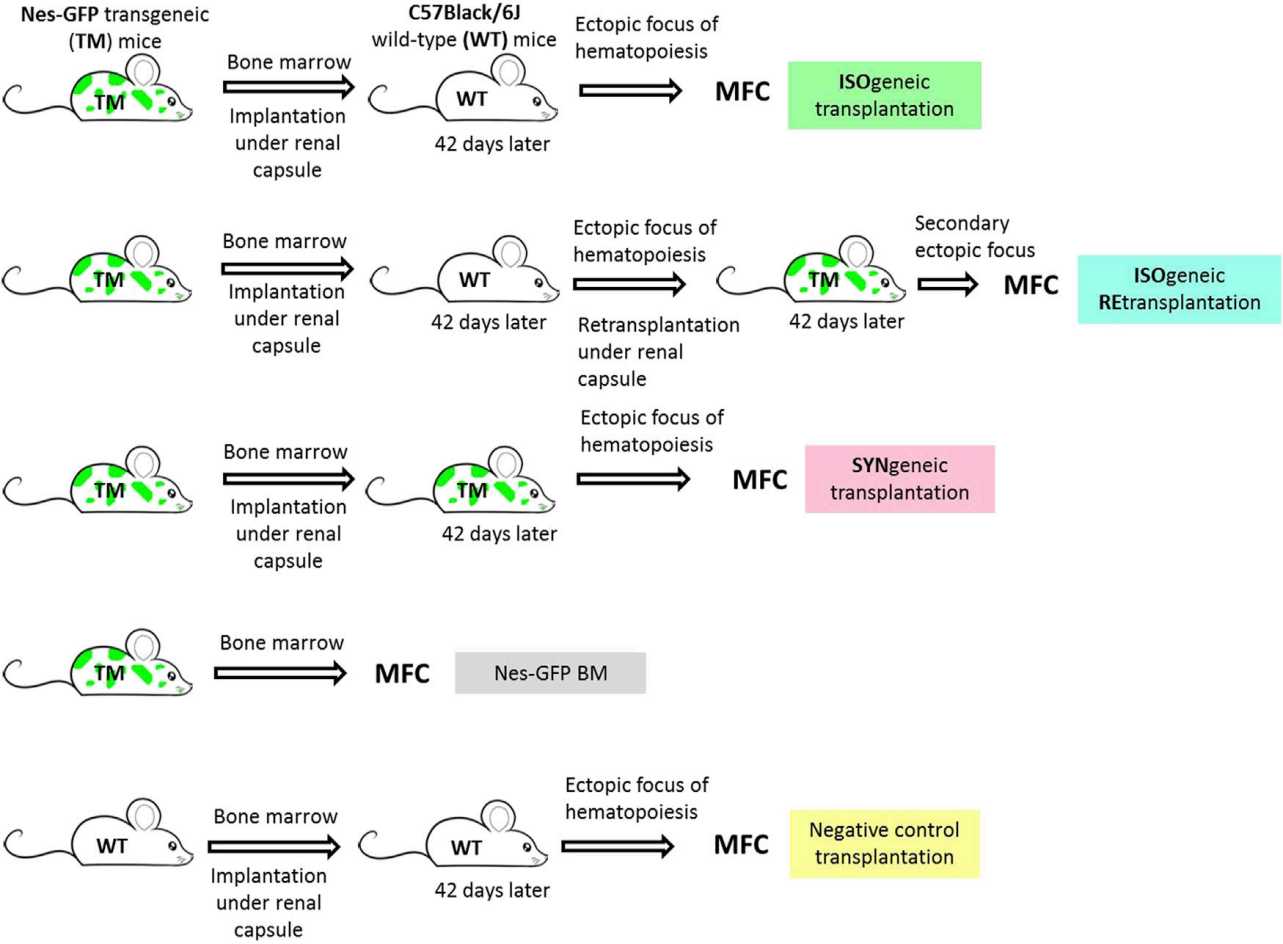
Experimental groups. Isogenic transplantation group corresponds to C57Black/6J wild-type immunocompetent recipients of Nes-GFP bone marrow under the renal capsule. Isogenic retransplantation group corresponds to Nes-GFP recipients of primary foci formed in isogneic transplantations. Syngenic transplantation group corresponds to Nes-GFP recipients of Nes-GFP bone marrow under the renal capsule. Nes-GFP BM group is primary bone marrow of Nes-GFP mice. Negative control transplantation corresponds to C57Black/6J wild-type immunocompetent recipients of syngenic bone marrow under the renal capsule.

BM of Nestin-GFP F1 (hereinafter referred as Nes-GFP) was implanted to C57Black/6J wildtype (WT) mice (*n* = 18). These were considered to be isogenic transplantations. As a positive syngenic control, we implanted the BM of one femur (*n* = 8) of Nes-GFP to Nes-GFP recipients (*n* = 8) (hereinafter syngenic transplantations). As a negative control, we implanted BM of one femur of C57Black/6J (*n* = 8) to C57Black/6J recipients (*n* = 5). Some of the primary foci formed in isogenic C57Black/6J recipients (*n* = 7) were retransplanted into the secondary Nes-GFP recipients (*n* = 7) (hereinafter isogenic retransplantations). As a positive syngenic control, we retransplanted one primary positive control to the secondary syngenic Nes-GFP recipient (hereinafter syngenic retransplantations). Experimental groups are depicted in Figure 2.

### Multicolor flow cytometry

BM from femurs and/or shins was resuspended in PBS, and the cells were counted in an Abacus Junior 30 hematology analyzer. Erythrocytes were lysed with PharmLyse solution (BD biosciences, United States) according to manufacturer instructions. No more than 4 × 10^6^ cells were incubated for 20 min in the dark with anti-CD45 monoclonal antibodies labeled with APC (eBiosciences, United States), 7-AAD (Sigma Aldrich, United States) and Syto41 (Invitrogen, United States). 7-AAD was added to exclude dead cells; Syto41 was added to exclude enucleated cells. The analysis was performed using a CytoFlex flow cytometer (Beckman Coulter, United States); the data were analyzed using the Kaluza Analysis v. 2.1 (Beckman Coulter, United States). The gating strategy is given in the Supplementary Material (Supplementary Figure S3).

### Inclusion criteria

We set three inclusion criteria for samples for data presentation. First, we visually assessed the site of implantation and did not take into account apparently flawed samples. These ones included samples, in which only residual scars or pigmentation or an ossicle without red internal cell mass were observed at the site of operation. Next, we excluded foci, in which the number of alive nucleated cells was less than 50,000. Thirdly, we suggested the proportion of hematopoietic and non-hematopoietic cells reflects the right cellular composition of the focus and is related to its functionality. We created a rank order plot for the ratio of CD45^+^ to CD45^–^ cells and graphically determined the range of acceptability as 43—241 (see Supplementary Material for details). This was in line with the observation that this ratio was distorted in case of flawed foci.

### Statistical analysis

Data for a proportion of target cells were processed on a log scale. If no target cells were detected, we assumed that the proportion of target cells was less than a detection limit, which was individual for each sample and was set to be one cell per number of alive nucleated cells in the sample. For the convenience of calculations, we set the proportion of target cells in such samples equal to 0.1 of the detection limit. The results are presented as geometric mean ×÷ geometric standard deviation factor. The comparisons between experimental groups were performed by the Mann-Whitney test.

## Results

Primary foci of ectopic hematopoiesis were found in 96% of cases (n = 23/24 foci in 12 recipients, including a pilot experiment) 42 days after the implantation of Nes-GFP BM under the kidney capsule of WT individuals. In the pilot experiment, foci of ectopic hematopoiesis were found in 100% (n = 6/6 foci in three recipients) cases (see Supplementary Material for details). Increased level of autofluorescence in GFP detection channel did not allow us unambiguously establish the presence of GFP^+^ cells within the foci (Karpenko and Bigildeev 2021). However, we managed to overcome this obstacle in a further experiment by reducing jet strength during the separation of interior cells of a focus from its bone shell and by modifying the MFC analysis protocol. This protocol was evaluated on native BM of Nestin-GFP mice. In total, 10.6 × 10^–5^ ×÷1.2 of alive BM cells washed from the femur were GFP^+^CD45^–^ (Table 1), while the proportion of GFP^+^CD45^–^ cells in BM of Nestin-GFP mice was previously calculated as 80 × 10^–5^, as measured by MFC (Méndez-Ferrer et al., 2010).

**TABLE 1:** Characteristics of individual samples of experimental groups acoording to MFC analysis. * Zero cell counts were transformed into non-zero proportions as described in Materials and Methods to estimiate measurement accuracy.

Group	GFP^+^ cells Proportion, × 10^−5^; Number, cells				Alive nucleated cells in a sample
	CD45^–^		CD45^+^		
Nestin-GFP BM	13,15	*140*	79,64	*848*	1064738
	8,43	*173*	63,84	*1310*	2051921
	10,66	*52*	126,64	*618*	488000
syngenic transplantation	0,95	*6*	1,27	*8*	631375
	6,99	*24*	69,87	*240*	343493
	1,20	*8*	84,87	*565*	665705
	1,08	*13*	12,46	*150*	1203524
isogenic transplantation	16,06	*49*	2,62	*8*	305111
	4,53	*45*	14,50	*144*	993267
	< 0.02*	*0*	< 0.02*	*0*	615881
	6,95	*131*	15,75	*297*	1885924
	1,47	*22*	7,54	*113*	1497972
	4,29	*32*	2,68	*20*	745908
	2,09	*23*	1,72	*19*	1102013
isogenic retransplantation	14,70	*10*	110,27	*75*	68016
	10,11	*36*	56,16	*200*	356143
	2,64	*4*	144,06	*218*	151322
	3,27	*13*	162,69	*646*	397081
	10,89	*25*	158,08	*363*	229631
negative control transplantation	< 0.03*	*0*	< 0.03*	*0*	302269
	< 0.11*	*0*	< 0.11*	*0*	89398
	< 0.01*	*0*	< 0.01*	*0*	779434
	< 0.15*	*0*	< 0.15*	*0*	67475

**FIGURE 3:**
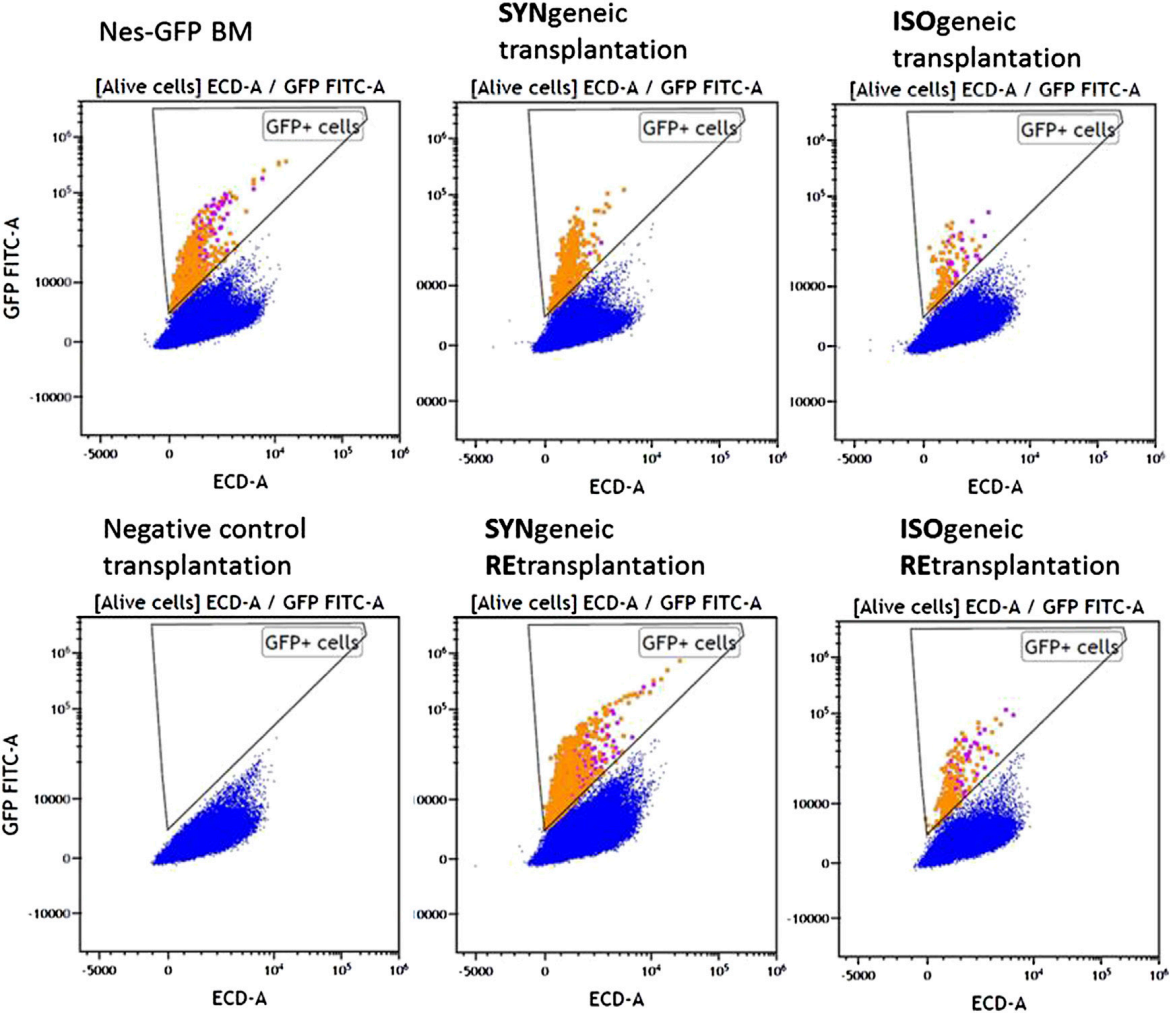
MFC data for representative samples of experimental groups. Alive nucleated cells. Orange and purple dots correspond to GFP^+^CD45^+^ and GFP + CD45^–^ cells, respectively.

MFC analysis showed that 7/8 primary isogenic foci satisfied inclusion criteria. Similarly, the foci formed in 100% of cases after syngenic Nestin-GFP transplantations (n = 8), and four of them satisfied inclusion criteria. Each included focus had a normal bone shell. The presence of GFP^+^ cells both in CD45^–^ and CD45^+^ cell subpopulations was confirmed in 6/7 isogenic transplantations using MFC (Figure 3).

**FIGURE 4:**
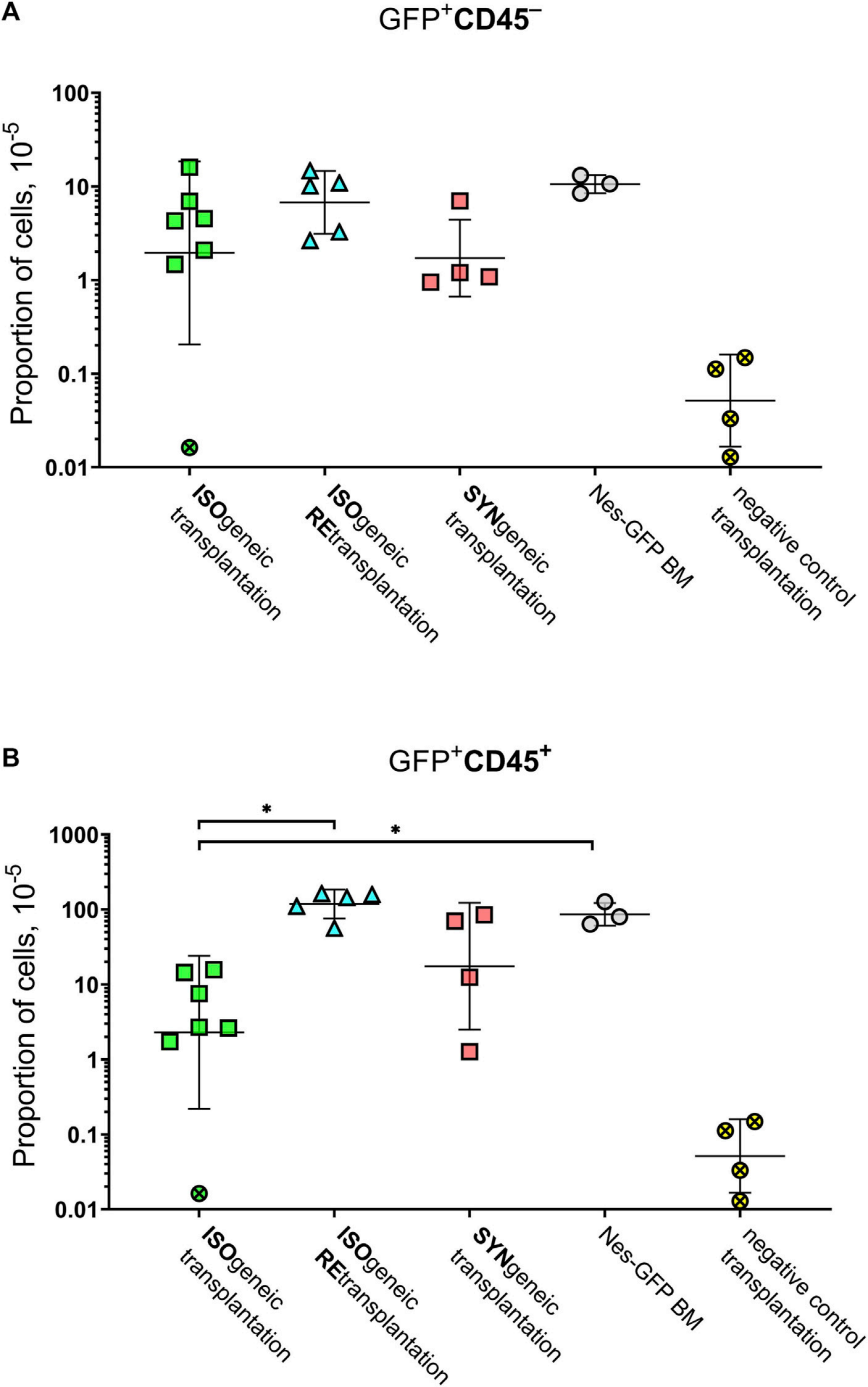
Graphical representation of the frequencies of GFP^+^ cells. **(A)** GFP^+^CD45^–^ cells. **(B)** GFP^+^CD45^+^ cells. Horizontal lines designate geometric mean values. Whiskers designate geometric standard deviation. Crossed markers designate samples, in which GFP^+^ cells were not detected. They were processed as described in Materials and Methods to estimate measurement accuracy.

GFP^+^CD45^–^ (Figure 4A) and GFP^+^CD45^+^ (Figure 4B) cells were detected in each focus from syngenic transplantations. Interestingly, the number of CD45^+^GFP^+^ cells was comparable to or even higher than the number of GFP^+^CD45^–^ cells (Table 1 and Figure 4). No GFP^+^ cells were detected in negative controls.

The mean proportion of GFP^+^ cells was 4.8 × 10^–5^ ×÷10 and 21 × 10^–5^ ×÷6 in isogenic and syngenic transplantations, respectively. The mean proportion of GFP^+^CD45^–^ cells was 2.0 × 10^–5^ ×÷9 and 1.7 × 10^–5^ ×÷3 in isogenic and syngenic transplantations, respectively. The mean proportion of GFP^+^CD45^+^cells was 2.3 × 10^–5^ ×÷10 and 17.5 × 10^–5^ ×÷7 in isogenic and syngenic transplantations, respectively.

Retransplantation of transgenic primary foci into Nestin-GFP secondary recipients was successful after prolonged exposure to the native immune system. Results of MFC demonstrated GFP^+^CD45^+^ and GFP^+^CD45^–^ cells in the secondary foci in numbers that were similar to such numbers for native Nestin-GFP BM or syngenic retransplantation (see Supplementary Table S2). Specifically, the proportion of GFP^+^CD45^–^ and GFP^+^CD45^+^ cells was 6.7 × 10^–5^ ×÷2.2 and 118 × 10^–5^ ×÷1.6 in isogenic retransplantations.

## Discussion

In the first few days after the BM implantation, the hematopoietic microenvironment ceases to function as a substrate that supports the proliferation and differentiation of HSCs (Chertkov and Gurevitch 1980). During the first 2 days after implantation, necrosis occurs onsite, leaving behind a reticular network of adventitial cells (Tavassoli and Crosby 1968), which then proliferate and differentiate to give rise to a trabecular bone. It is almost completely resorbed afterward except for a thin bone shell underneath the renal capsule. After 5–6 weeks, a new fully functional extramedullary focus of hematopoiesis is formed, which is not distinguishable from normal BM in cellular and morphological aspects and response to external stimuli (Chertkov et al., 1983a; Sadr, Cardenas, and Tavassoli 1980; Tavassoli and Crosby 1968; Friedenstein et al., 1968). This, in turn, means the presence of a full-fledged microenvironment with the correct architectonics and the presence of the necessary cellular elements of the stroma in the required proportions. The stroma of such focus belongs to the donor, and the hematopoietic cells represent a mix of donor and recipient cells (Chertkov et al., 1983b). It is considered that the hematopoietic microenvironment is built *de novo* as a result of the proliferation and differentiation of MSCs (Chertkov and Gurevitch 1980). We used this method by implanting BM of Nes-GFP under the renal capsule of the C57Black/6J strain. The strength of immunologic responses to GFP in C57Black/6J is weaker than in BALB/c, but several studies show the clearance of GFP^+^ cells upon transplantation, and the immunodominant CTL epitope of GFP is identified in C57Black/6J mice (Stripecke et al., 1999; Rosenzweig et al., 2001; Han, Unger, and Wauben 2008; Uchida et al., 2014; Sats et al., 2015; Ansari et al., 2016).

Although MSCs contained an immunogenic marker, full-fledged primary foci of ectopic hematopoiesis initiated from Nes-GFP BM under the kidney capsule of WT mice formed. Moreover, the secondary foci also formed. This means that MSCs fulfill their functions of building hematopoietic territory. GFP^+^ cells found in isogenic foci are immunoprivileged.

Part of immunoprivileged GFP^+^ cells within the isogenic foci was CD45^–^. The fact that statistically significant difference was not found (*p* = 0.41) in the proportion of such cells between syngenic and isogenic foci and also the evidence that the proportion of such cells was similar (*p* = 0.79) in Nes-GFP BM and isogenic retransplantations argue in favor that the subpopulation CD45^–^GFP^+^ is not suppressed by the recipient’s immunity in an isogenic transplant.

Isogenic retransplantation showed the formation of the secondary foci after a prolonged stay (42 days) of MSCs in the primary recipient under the exposure to immunity. This suggests that the function of immunoprivileged MSCs, namely their ability to form and maintain the hematopoietic territory *in vivo*, is preserved. The proportion of GFP^+^CD45^–^ cells in secondary foci turned out to be even slightly higher than in primary foci for both syngenic and isogenic retransplantations, which indicates the ability of these cells for self-maintenance and expansion *in vivo*. This ability remains even after prolonged exposure to the entire immune system in case of isogenic retransplantations.

By combining the results of published studies showing that MSCs in Nestin-GFP mice express GFP (Méndez-Ferrer et al., 2010; Nobre et al., 2021) with our results showing preserved MSCs function in isogenic retransplantation, we conclude that MSCs are immunoprivileged cells among observed GFP^+^ cells.

We suggest that the successful demonstration of MSCs’ IPs in this study in contrast to those studies, which could not demonstrate the phenomenon (Badillo et al., 2007; Zangi et al., 2009; Miura et al., 2013; Ankrum, Ong, and Karp 2014; Berglund et al., 2017), may follow from several key points (Figure 1). The first one comes from untangling the confusion between notions of *in vivo* existing MSCs and *in vitro* propagated stromal cells named multipotent mesenchymal stromal cells (MMSCs) (Horwitz et al., 2005; Dominici et al., 2006; Caplan 2017). MMSCs are considered to be descendants of MSCs and may share some characteristics of MSCs. For example, the immunomodulatory properties of MMSCs were demonstrated (Le Blanc et al., 2004; Pradier et al., 2011), which resulted in their usage in the clinic to suppress various immune responses (Kuzmina et al., 2012; Auletta, Deans, and Bartholomew 2012; Mazzini et al., 2012; Sun et al., 2009). However, initial enthusiasm and hopes for MMSCs as an easily accessible allogenic but immune transparent therapeutic approach were hampered due to later studies, which stated that MMSCs are not immune-privileged cells and documented specific cellular and humoral immune responses against donor antigens following allo-MMSCs administration (reviewed in (Berglund et al., 2017)). These circumstances made a contribution to a transition to cell-free therapy basing on MMSC-derived exosomes (Mendt, Rezvani, and Shpall 2019). Also, some authors use alternative criteria for the definition of MSCs, such as the specific location within BM, their ability to support HSCs (Kunisaki 2019), or immunophenotype characteristics (Morikawa et al., 2009; Méndez-Ferrer et al., 2010; Omatsu et al., 2010; Armulik, Genové, and Betsholtz 2011; Kunisaki et al., 2013; Baryawno et al., 2019). The second key point is that we used a model, where most transplanted cells were immunologically identical to the recipient except for a small part of cells associated with Nestin^+^ stem/progenitor cells, and particularly Nestin^+^ MSCs, for which all described criteria for mesenchymal stem cells were fulfilled (Méndez-Ferrer et al., 2010) (Figure 1), while in the majority of other relevant studies, all injected cells were allogenic. The third key point is using unfractionated and minimally disturbed BM in our transplantations since it is known that dissociation of MSCs, as well as HSCs from their microenvironment, adversely affects their function and that local regulation is important for retention of stem cells in a quiescent state and implementation of IPs by them (Chertkov et al., 1983a; Nie, Han, and Zou 2008; Greenbaum et al., 2013; Boyd et al., 2014; Agudo et al., 2018). The site of administration of the cells or the local microenvironment may also play a role. For example, it was shown that upon the injection of suspensions of allogenic MSCs into the bone cavity in mice and the intraosseous administration of allogenic MMSCs in humans a long-term presence of allogenic cells at the injection site was observed (Cai et al., 2008; Kuzmina et al., 2016). However, these studies were carried out on recipients, who were immunosuppressed at the time of cell injection, and the stem cell status of survived cells was not verified. There are modifications to the model of ectopic foci formation, although they are less physiological. Chan with coauthors demonstrated that dissociated progenitor cells sorted from 15.5 days post-coitum mouse fetal bones and injected under the kidney capsule formed the foci (Chan et al., 2009). Additionally, Mendez-Ferrer with colleagues showed that sorted from the BM of adult animals Nes-GFP^+^ cells formed mesenspheres *in vitro*, which formed hematopoietic territory upon subcutaneous transplantation on phosphocalcic ceramic ossicles (Méndez-Ferrer et al., 2010).

We believe that the simultaneous consideration of these key points in this study made it possible to demonstrate the immune privileges of MSCs of BM. At the same time, the deviation from one or several of these positions at once was probably the reason for the impossibility of observing immune privileges by other authors (Zangi et al., 2009). Most of these studies used preliminary *in vitro* cultivated BM-derived stromal progenitor cells, which were dissociated and converted to single-cell suspension before injection and were all allogenic to the recipient. For example, one study used Balb/c murine adult bone marrow-derived stromal progenitor cells (AmSPCs), which were cultivated for 13 passages *in vitro*, then the cells were trypsinized and the single cell suspension was created, which was injected into the peritoneal cavity of C57Black/6J recipients (Badillo et al., 2007). The authors concluded that AmSPCs are recognized by the host immune system *in vivo*, elicit a cellular and humoral immune response, and fail to induce tolerance. However, the authors did not directly show the clearance of allogenic donor cells and they admit that AmSPCs may set up local immune suppressive environments after immune recognition allowing their persistence in host tissues.

It is known that Nestin-GFP^bright^CD45^–^Ter119^–^CD31^–^ cells of BM are a rare cell population, which is found exclusively along arterioles, account for the most CFU-F activity, and regulate the quiescent state of HSC. Moreover, Nestin-GFP^bright^CD45^–^Ter119^–^CD31^–^ cells are quiescent themselves (Kunisaki et al., 2013). Due to the high autofluorescene of cells isolated from the foci, we did not manage to unambiguously determine the gate for GFP^bright^ cells, however, due to the same reason we expectedly to detect brighter part of GFP^+^ cells. We support the idea that the ability to escape from immune clearance may be a common feature of slowly cycling adult mammalian stem cells (Agudo et al., 2018; Hirata et al., 2018), however more direct pieces of evidence are required.

GFP^+^CD45^+^ cells found in isogenic transplantations are also immunoprivileged. The presence of GFP^+^CD45^+^ hematopoietic cells in the BM of Nes-GFP adult mice was previously shown in several studies (Ludin et al., 2012; Itkin et al., 2016). CD45 is known to be expressed on a wide range of hematopoietic cells, including HSCs (Hermiston, Xu, and Weiss 2003; Dykstra et al., 2006; Shivtiel et al., 2008). However, the proportion of GFP^+^CD45^+^ in both primary and secondary isogenic transplantations significantly exceeds the proportion of HSCs in BM, for which IPs have been demonstrated previously, so the nature of detected cells requires further investigations. In the case of isogenic transplantation, the reduced proportion of GFP^+^CD45^+^ cells in the focus, which is observed when comparing isogenic and syngenic transplantation, may be since the hematopoietic cells of the recipient that populate the focus do not contain the transgene, and the proportion of recipient hematopoietic cells within the focus is known to be in the range 25%—93% (Chertkov et al., 1983b).

We cannot exclude the possibility that a part of GFP^+^ cells detected in isogenic transplantations could be non-immunoprivileged cells. They could partially represent macrophages that phagocyted GFP-producing cells. Another possible reason to see an expanded number of GFP^+^ cells is their location within niches, in which immune reactions are suppressed by mesenchymal stromal progenitors (Aldrich et al., 2021; Sergeant et al., 2021). Another explanation is that GFP^+^ cells partially may be descendants of immunoprivileged cells, for which the interplay between dynamics of the loss of GFP and IPs over the immune system allows their observation. These assumptions could account for a relatively high concentration of GFP^+^CD45^+^ cells.

In addition to MSCs, for which we functionally demonstrate IPs in this study, and to HSCs, for which IPs were reported previously, perhaps a part of immunoprivileged GFP^+^ cells could be different, for which IPs were not described earlier. For example, GFP^+^ cells are described among CD45^–^CD31^+^ endothelial cells in BM of adult Nes-GFP mice (Itkin et al., 2016). Interestingly, nestin is expressed in endothelial progenitor cells but not in mature endotheliocytes (Suzuki et al., 2010). GFP^+^CD45^–^ fraction of BM cells from Nes-GFP mice can also contain Gfap^–^ Schwann cell precursors (Isern et al., 2014). In another study with Nes-GFP mice, it was demonstrated that GFP^+^ cells can circulate in the peripheral blood and accumulate in the pulmonary tissue during the chronic phase of infection in mice (Coimbra-Campos et al., 2021). The authors note that most of these cells co-express the CD45 marker. Thus, demonstrated IPs may extend to several cell populations other than MSCs and HSCs in murine BM.

Nestin expression is characteristic of a wide range of stem/progenitor cells found in different tissues and organs over the whole body not only in mice but in humans, as well (Bernal and Arranz 2018). Nestin is also associated with CSCs of different tissue origins and poor survival prognosis (Neradil and Veselska 2015). It is tempting to extrapolate that a wide range of immunoprivileged cells in an adult mammalian is nestin-positive. Nestin is expressed by quiescent stem cells in immune-privileged organs, such as the testis (Jiang et al., 2014), cartilage (Jaramillo-Rangel et al., 2021), brain (Li et al., 2003), and retina (Bhatia et al., 2009). Moreover, MuSCs and HFSCs are also characterized by GFP expression in Nestin-GFP adult mice (Li et al., 2003; Day et al., 2007). This means that nestin is a potential marker of immunoprivileged cells in the adult mammalian organism. In this aspect, the Nestin-GFP mouse strain is a convenient tool, which can be used to test IPs of a wide range of stem/progenitor cells. Currently, the link between nestin expression and IPs is unknown in the scientific literature, so summing up all of the above, we suggest the existence of such a link. Though, whether nestin is a passive marker of activation of other mechanisms of IPs or has a direct role in it requires further research.

## Conclusion

We conclude that MSCs of BM have IPs *in vivo*, at least in C57Black/6J mouse strain in ectopic foci of hematopoiesis setting. So, MSCs of BM, MuSCs, HFSCs, and HSCs are stem cells of different embryogenic origins, which can escape immunity. Since such cells can be isolated from almost any organ and tissue, the IPs may be spread throughout the mammalian body. The results of this study strengthen the idea that IPs are a common quality of quiescent stem cells. So, stem cells possess some important defensive mechanisms including resistance to genotoxic and cytostatic compounds, ionizing radiation, and IPs. These features are also characteristics of CSCs. They may be acquired by a cancer cell not only independently, but also simultaneously as a consequence of obtaining a stem cell state. This provides a valuable contribution to our understanding of oncogenesis. Recognition of MSCs as cells with IPs can further potentiate their usage for gene delivery and improvement of allogenic transplant engraftment. Our study emphasizes that niche integrity, as well as accessory cells, play role in the affordance and maintenance of IPs. We suggest the existence of a link between nestin expression and IPs, based on the synthesis of this study and others. This study offers a model and a novel view of Nestin-GFP mouse strain for studying IPs of a wide range of stem/progenitor cells in an adult mammalian organism and the interaction of BM stroma, in general, and MSCs, in particular, with the immune system *in vivo* under conditions close to native ones. This approach can be easily broadened to other mouse strains by crossbreeding of Nestin-GFP mice with the strain of interest and implanting F1 BM to the parent non-transgenic strain.

## Data Availability

The original contributions presented in the study are included in the article/Supplementary Material, further inquiries can be directed to the corresponding author.
