# Supplementary material for: Nestin-GFP transgene labels immunoprivileged bone marrow mesenchymal stem cells in the model of ectopic foci formation
Source: Front Cell Dev Biol. 2022 Sep 5;10:993056. doi: 10.3389/fcell.2022.993056 (PMC9483855; doi:10.3389/fcell.2022.993056)
Supplement: Supplementary file 1 [file DataSheet1.docx]

**Pilot experiment details**

Second-generation hybrids (F2) were obtained by crossbreeding F1 hybrids (Nestin-GFP × C57Black/6J) to obtain animals whose genotype differed only in the presence of the GFP transgene. F2 hybrids were used for the experiments at the age of 25-50 weeks. The presence or absence of the transgene in each F2 individual was determined by PCR for a region of *Gfp* gene in DNA isolated from a piece of the tail (Fig. S1). The BM of one femur (n = 6) of GFP^+^ F2 animal was used for implantation to the GFP^–^ littermate (n = 3). All other procedures were the same as in the main experiment. All the foci had a bone shell. The cellularity of the foci did not differ from the cellularity of the control syngeneic foci (data not shown). In the foci obtained from the Nes-GFP^+^ F2 BM, the formation of a full-fledged bone shell, which visually did not differ from syngeneic controls, was observed.

**CD45+/CD45- inclusion criterion**

We suggested the proportion of hematopoietic and non-hematopoietic cells reflects right cellular composition of the focus and is related to its functionality. To take this into account we created rank order plot for the ratio of CD45^+^ to CD45^–^ alive nucleated cells (Fig. S2). Here we took into account all samples from all groups without any exceptions. Basing on the plot, we excluded samples that corresponded to peripheral inflections. Graphically determined range of acceptability was arbitrarily set as 43 – 241.


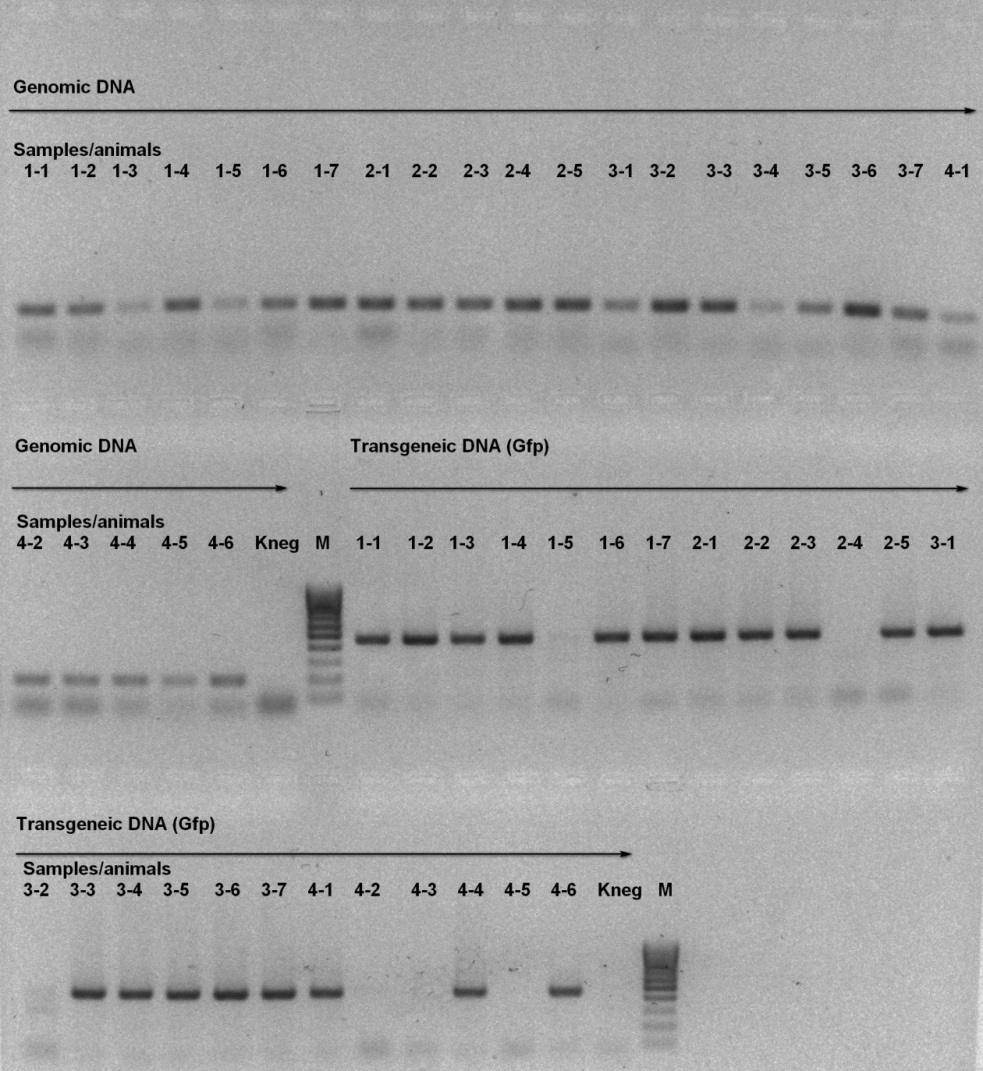


Fig. S1. Genotyping of F2 hybrids. Animals 2-4, 4,2, 4-3, and 4-5 were considered to be GFP–/–, animal 3-2 was excluded from the experiment as putative, the rest animals were considered to be GFP+. M – Molecular length marker, 100 bp ladder; Kneg – negative PCR control

Fig. S2. Rank order plot for the ratio of CD45^+^ to CD45^–^ cells. Black circles correspond to samples that were included in analysis; white circles correspond to samples that were excluded.


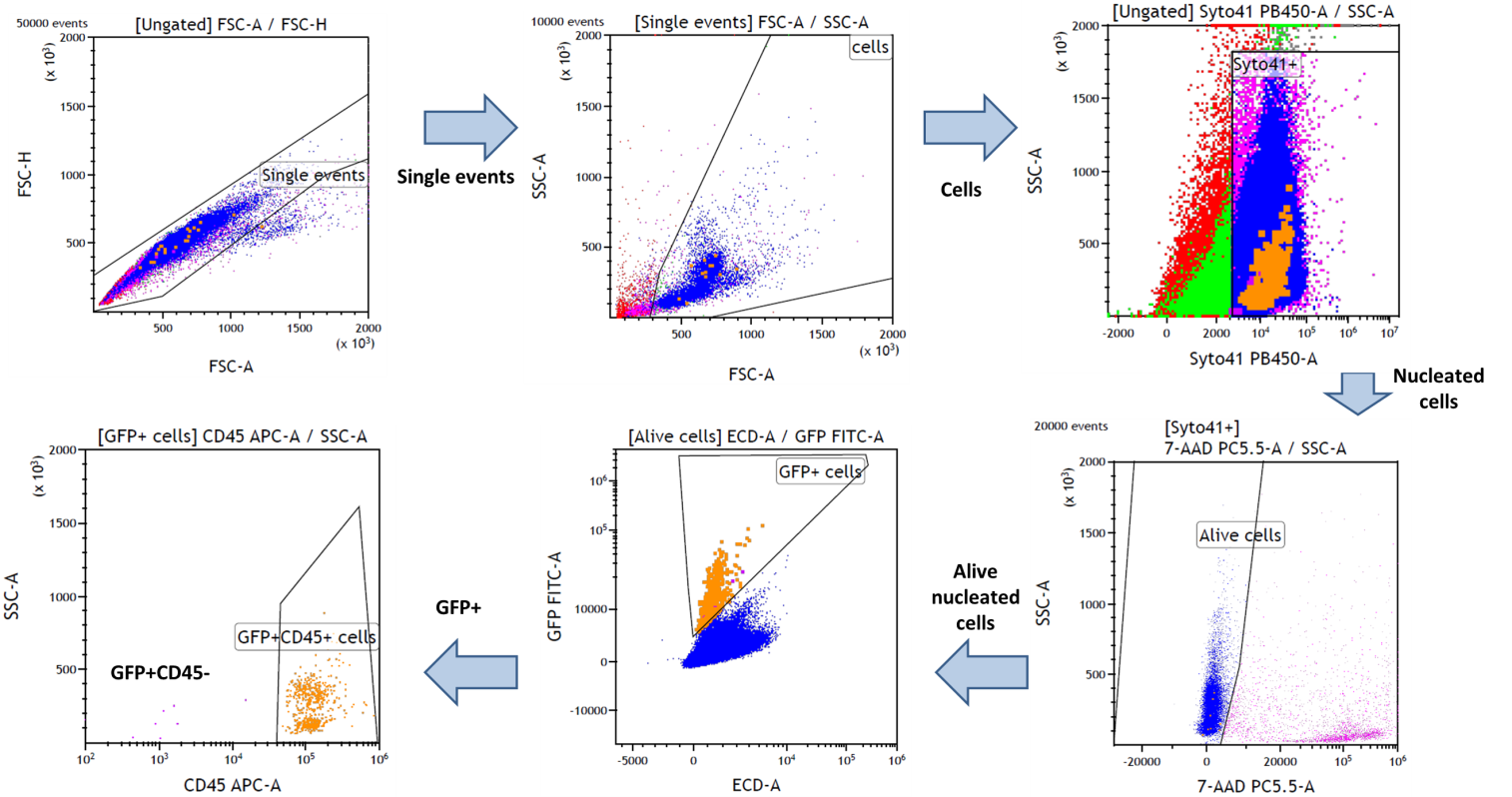


Fig. S3. MFC gating strategy.

Table S1. Primers and probes used in the experiment

| Name | Sequence |
| --- | --- |
| EGFP-w1 | ATGGTGAGCAAGGGCGAGGA |
| EGFP-C1 | AGACGTTGTGGCTGTTGTAG |
| Actb_mm_DNA_F | GTGACCTGTTACTTTGGGAG |
| Actb_mm_DNA_R | GTCCCAGTTGGTAACAATGC |

Table S2. Characteristics of Nes-GFP ectopic foci in C57Black/6J F1 recipients. * Zero cell counts were transformed into non-zero proportions as described in Materials and Methods to estimiate measurement accuracy

| Group | GFP^+^ cells  **Proportion,** × **10^-5^**; *Number, cells* | | | | Alive nucleated cells in a sample |
| --- | --- | --- | --- | --- | --- |
|  | CD45^–^ | | CD45^+^ | |  |
| Nestin-GFP BM | **13,15** | *140* | **79,64** | *848* | 1064738 |
|  | **8,43** | *173* | **63,84** | *1310* | 2051921 |
|  | **10,66** | *52* | **126,64** | *618* | 488000 |
| syngeneic transplantation | **0,95** | *6* | **1,27** | *8* | 631375 |
|  | **6,99** | *24* | **69,87** | *240* | 343493 |
|  | **1,20** | *8* | **84,87** | *565* | 665705 |
|  | **1,08** | *13* | **12,46** | *150* | 1203524 |
| isogeneic transplantation | **16,06** | *49* | **2,62** | *8* | 305111 |
|  | **4,53** | *45* | **14,50** | *144* | 993267 |
|  | **< 0.02*** | *0* | **< 0.02*** | *0* | 615881 |
|  | **6,95** | *131* | **15,75** | *297* | 1885924 |
|  | **1,47** | *22* | **7,54** | *113* | 1497972 |
|  | **4,29** | *32* | **2,68** | *20* | 745908 |
|  | **2,09** | *23* | **1,72** | *19* | 1102013 |
| isogeneic retransplantation | **14,70** | *10* | **110,27** | *75* | 68016 |
|  | **10,11** | *36* | **56,16** | *200* | 356143 |
|  | **2,64** | *4* | **144,06** | *218* | 151322 |
|  | **3,27** | *13* | **162,69** | *646* | 397081 |
|  | **10,89** | *25* | **158,08** | *363* | 229631 |
| syngeneic retransplantation | **6,00** | *72* | **118,39** | *1420* | 1199392 |
| negative control transplantation | **< 0.03*** | *0* | **< 0.03*** | *0* | 302269 |
|  | **< 0.11*** | *0* | **< 0.11*** | *0* | 89398 |
|  | **< 0.01*** | *0* | **< 0.01*** | *0* | 779434 |
|  | **< 0.15*** | *0* | **< 0.15*** | *0* | 67475 |
